# Complete chloroplast genome sequence data of *Morus nigra* L.

**DOI:** 10.1016/j.dib.2026.113362

**Published:** 2026-10-22

**Authors:** Binbin Wang, Danyang Wang, Hui Wang, Yanxia Gao

**Affiliations:** a Institute of Sericulture, Chengde Medical University, Chengde, 067000, China; b Hebei Sericulture Industry Technology Innovation Center, Chengde, 067000, China

**Keywords:** Chloroplast genome, *Morus nigra* L., Phylogenetic tree, Microsatellites

## Abstract

*Morus nigra* L. is a black mulberry species with high medicinal and ornamental value. Its complete chloroplast genome was obtained using a combination of Illumina and Oxford Nanopore high-throughput sequencing platforms. The circular chloroplast genome of *M. nigra* is 159,330 bp in length with a GC content of 36.15%. It consists of a large single-copy (LSC) region of 88,104 bp, a small single-copy (SSC) region of 19,870 bp, and two inverted repeat (IR) regions (IRa and IRb) of 25,678 bp each. The chloroplast genome contains 130 genes, including 85 protein-coding genes, 37 transfer RNA (tRNA) genes, and eight ribosomal RNA (rRNA) genes. A total of 451 simple sequence repeat (SSR) loci were identified, among which dinucleotide repeats were the most dominant (219 loci, 48.6%). Phylogenetic analysis based on the chloroplast genomes of 27 species showed that all Morus species formed a monophyletic clade, with *M. nigra* occupying an independent branch. These data expand the genetic resources available for *Morus* and provide a foundation for future studies on the development and utilization of this species.

Specifications Table

**Table:** 

Subject	Biology
Specific subject area	Omics: Genomics*.*
Type of data	Table, Figure Raw, Analyzed.
Data collection	The chloroplast genome of *Morus nigra* L. was sequenced using Illumina Novaseq 6000 and Oxford Nanopore platform*.*
Data source location	City: Chengde City, Hebei Province Country: China Coordinates: 41°2′19.40″N, 117°57′48.10″E The voucher specimen was deposited at the Sericultural Research Institute of Chengde Medical University (http://cys.cdmc.edu.cn/, contact: Binbin Wang, wbb232456@cdmc.edu.cn, voucher number: CYYJS01)*.*
Data accessibility	Repository name: NCBI (National Center for Biotechnology Information) Data identification number: ON262114 Direct URL to data: https://www.ncbi.nlm.nih.gov/nuccore/ON262114.1/ The associated BioProject, SRA, and Bio-Sample numbers are PRJNA856852, SRR20303319, SRR20303320, and SAMN29594311, respectively. Repository name: Mendeley Data Data identification number: doi:10.17632/pbmpwhh3kz.1 Direct URL to data: https://data.mendeley.com/datasets/pbmpwhh3kz/1
Related research article	‘none’.

## 1 Value of the Data

• This dataset adds the complete chloroplast genome of *Morus nigra* L. to the growing collection of *Morus* plastomes reported in previous studies [1,2], providing a species-specific reference for this biologically distinctive and extremely high-polyploid lineage (2n = 22x = 308) [3].
• The phylogenetic and genomic data place *M. nigra* within the broader context of *Morus* diversity and provide a useful resource for comparative analyses of maternal lineage divergence and the evolutionary relationships among mulberry species.
• The annotated chloroplast genome and SSR markers generated in this study provide molecular tools for species identification, germplasm evaluation, and future studies of genetic diversity and interspecific hybridization in *Morus*.

## 2 Background

*Mulberry* (*Morus spp*.) has been cultivated for thousands of years and is widely distributed worldwide. Owing to its abundant bioactive secondary metabolites, mulberry fruits, roots, and stem bark have been used in folk medicine to treat a variety of conditions, including diabetes, hypotension, anemia, and arthritis [4,5]. *Morus nigra* L., commonly known as black mulberry, originated in western Iran and was introduced into the Xinjiang Uyghur Autonomous Region of China in the sixteenth century (Fig. 1). This species occupies a unique position among flowering plants because it has a chromosome number of 2n=308 (22x), the highest reported for any known species [3]. In addition, *M. nigra* is used in traditional Uyghur medicine and contains diverse compounds, including anthocyanins, phenols, and flavonoids, which exhibit antioxidant, anti-inflammatory, antitumour, and antibacterial activities [6,7].

**Fig. 1:**
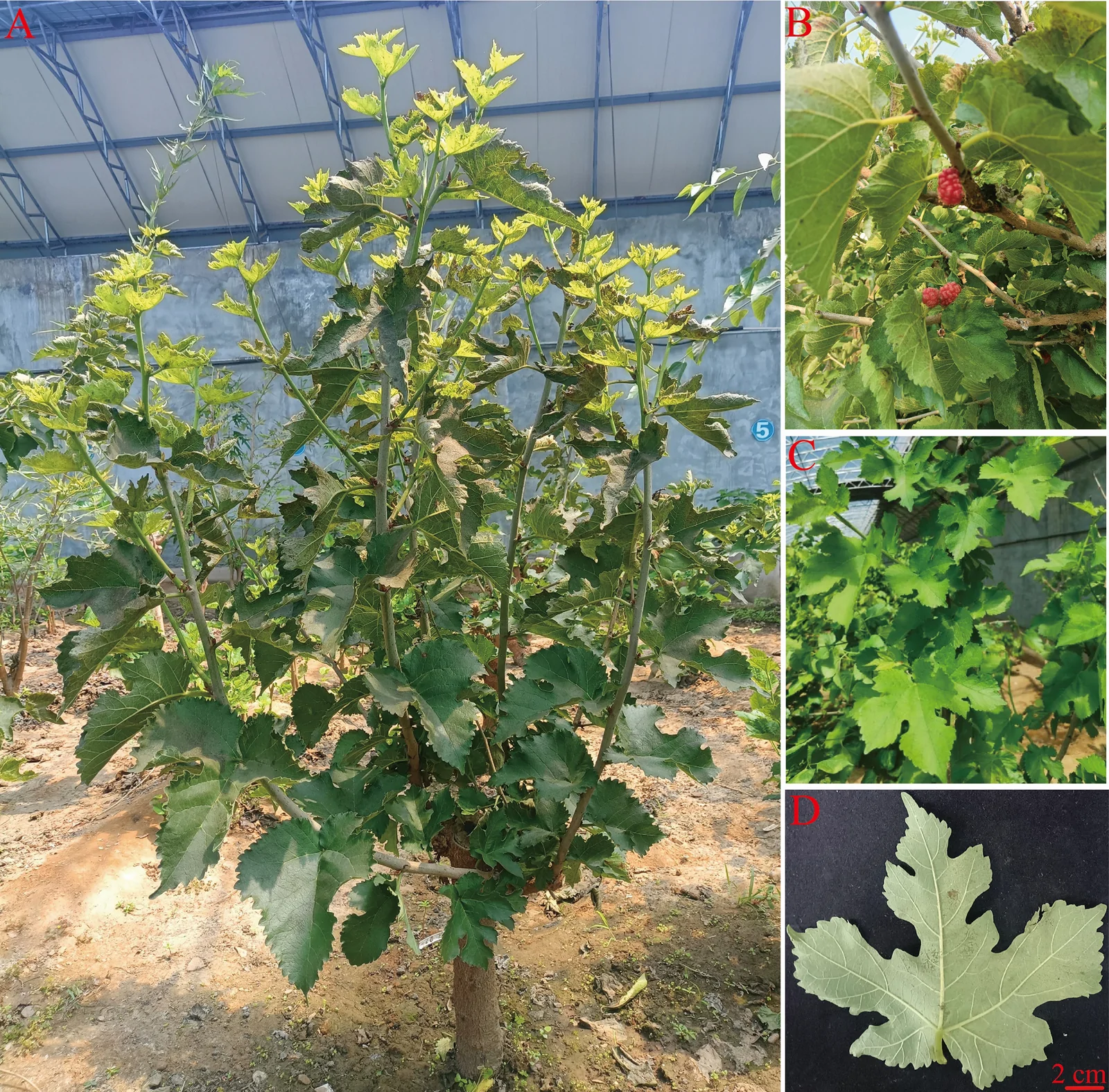
Morphology of *Morus nigra*. A. Whole plant. B. Stem and fruits. C, D. Divided leaves.

Morphological and molecular phylogenetic analyses have previously been used to classify *Morus* species [8,9]. Although the monophyly of the genus is well supported, its internal taxonomy remains unresolved because of extensive transplantation, interspecific hybridization, and introgression. Compared with the nuclear genome, the chloroplast genome is small, structurally simple, typically uniparentally inherited, and highly conserved in gene content and sequence, making it a useful tool for species identification and evolutionary inference [10,11]. At present, complete chloroplast genomes from multiple *Morus* species have been reported [1,2]. However, studies on *M. nigra* have largely focused on its nuclear genome, and its chloroplast genome remains poorly characterized. In this study, we sequenced and assembled the chloroplast genome of *M. nigra* using Illumina NovaSeq 6000 and Oxford Nanopore platforms, analyzed its structural features, and constructed a phylogenetic tree using representative Moraceae species to infer its evolutionary relationships. The data provide a theoretical basis for future genetic improvement and evolutionary studies.

## 3 Data Description

In this study, the chloroplast genome of *M. nigra* was 159,330 bp in length and exhibited the typical quadripartite structure, comprising a large single-copy (LSC) region (88,104 bp), a small single-copy (SSC) region (19,870 bp), and two inverted repeat (IR) regions (IRa and IRb) of 25,678 bp each (Fig. 2; Table 1). The genome contained 130 genes, including 85 protein-coding genes, 37 tRNA genes, and eight rRNA genes. The IR regions contained 18 duplicated genes, including four rRNA genes (*rrn23S, rrn4.5S, rrn5S*, and *rrn16S*), seven tRNA genes (*trnA-UGC, trnI-CAU, trnI-GAU, trnL-CAA, trnN-GUU, trnR-ACG*, and *trnV-GAC*), and seven protein-coding genes (*rpl2, rpl23, rps7, rps12, ndhB, ycf1*, and *ycf2*). The LSC region contained 60 protein-coding genes and 22 tRNA genes, whereas the SSC region contained 11 protein-coding genes and one tRNA gene.

**Fig. 2:**
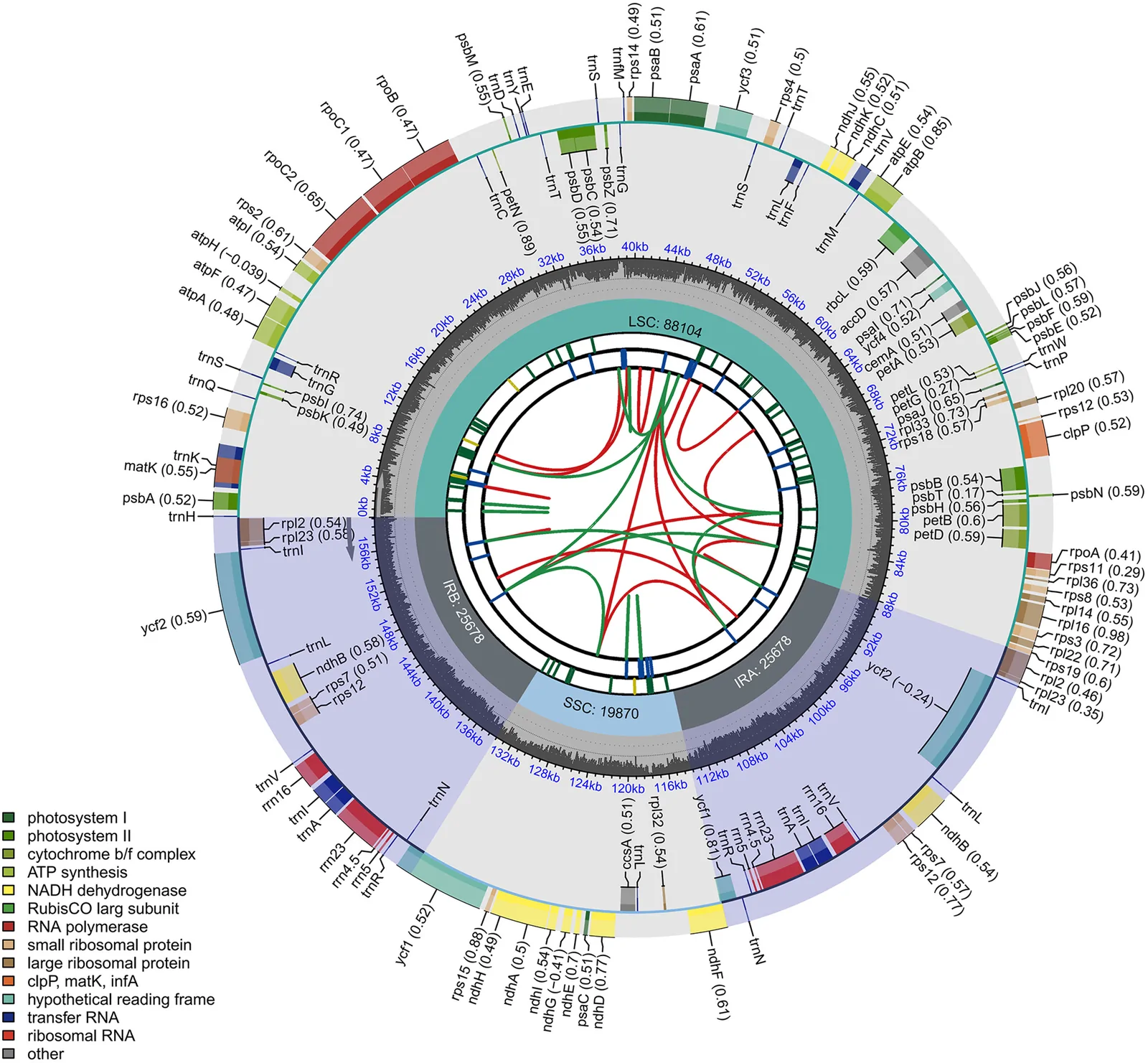
Circular map of the chloroplast genome of *M. nigra*.

**Table 1:** Functional classification of genes annotated in the chloroplast genome of *M. nigra*.

Groups of Genes	Genes	Number
Protein-coding genes		
ATP synthase	*atpA, atpB, atpE, atpF*⁎, *atpH, atpI*	6
Cytochrome b/f complex	*petA, petB*⁎, *petD*⁎, *petG, petL, petN*	6
RubisCO large subunit	*rbcL*	1
NADH dehydrogenase	*ndhA*⁎, *ndhB*⁎ (×2), *ndhC, ndhD, ndhE, ndhF, ndhG, ndhH, ndhI, ndhJ, ndhK*	12
Photosystem I	*psaA, psaB, psaC, psaI, psaJ*	5
Photosystem II	*psbA, psbB, psbC, psbD, psbE, psbF, psbH, psbI, psbJ, psbK, psbL, psbM, psbN, psbT, psbZ*	15
Hypothetical chloroplast reading frame (ycf)	*ycf1* (×2), *ycf2* (×2), *ycf3*⁎⁎, *ycf4*	6
Ribosomal proteins		
Large subunits	*rpl2*⁎ (×2), *rpl14, rpl16, rpl20, rpl22, rpl23* (×2), *rpl32, rpl33, rpl36*	11
Small subunits	*rps2, rps3, rps4, rps7* (×2), *rps8, rps11, rps12*⁎⁎(×2), *rps14, rps15, rps16*⁎, *rps18, rps19*	14
RNA polymerase	*rpoA, rpoB, rpoC1*⁎, *rpoC2*	4
Acetyl-CoA carboxylase	*accD*	1
Inner envelope membrane	*cemA*	1
Cytochrome c biogenesis protein	*ccsA*	1
Protease	*clpP*⁎⁎	1
Maturase	*matK*	1
RNA Genes		
Ribosomal RNAs	*rrn4.5S* (×2), *rrn5S* (×2), *rrn16S* (×2), *rrn23S* (×2)	8
Transfer RNAs (tRNAs)	*trnA-UGC*⁎ (×2), *trnC-GCA, trnD-GUC, trnE-UUC, trnF-GAA, trnfM-CAU, trnG-GCC, trnG-UCC*⁎, *trnH-GUG, trnI-CAU* (×2), *trnI-GAU*⁎ (×2), *trnK-UUU*⁎, *trnL-CAA* (×2), *trnL-UAA*⁎, *trnL-UAG, trnM-CAU, trnN-GUU* (×2), *trnP-UGG, trnQ-UUG, trnR-ACG* (×2), *trnR-UCU, trnS-GCU, trnS-GGA, trnS-UGA, trnT-GGU, trnT-UGU, trnV-GAC* (×2), *trnV-UAC*⁎, *trnW-CCA, trnY-GUA*	37

⁎ Indicates a gene containing one intron.

⁎⁎ Indicates a gene containing two introns; x2 indicates duplicated genes.

Fifteen genes contained a single intron (*rps16, atpF, rpoC1, petB, petD, rpl16, rpl2, ndhB, ndhA, trnK-UUU, trnG-UCC, trnL-UAA, trnV-UAC, trnI-GAU*, and *trnA-UGC*), and three genes (*ycf3, clpP*, and *rps12*) contained two introns. The overall GC content of the chloroplast genome was 36.15%, with GC contents of 33.79% in the LSC region, 29.14% in the SSC region, 42.92% in the IR regions (Table 2).

**Table 2:** Structural features of the chloroplast genome of *M. nigra*.

Region name	Start	End	Length (bp)	GC content (%)
LSC	1	88,104	88,104	33.79
IRa/IRb	88,105	113,782	25,678	42.92
SSC	113,783	133,652	19,870	29.14
IR	133,653	159,330	25,678	42.92

A total of 451 simple sequence repeat (SSR) loci were identified. Dinucleotide repeats were the most abundant (219 loci, 48.6%), followed by mononucleotide repeats (147 loci, 32.6%), tetranucleotide repeats (42 loci, 9.3%), trinucleotide repeats (30 loci, 6.7%), and pentanucleotide repeats (13 loci, 2.9%) (Fig. 3A). Among dinucleotide SSRs, AA/TT was the most common motif (146 loci, 32.1%), followed by AT/AT (51 loci, 11.2%) and AG/CT (21 loci, 4.6%). Among mononucleotide SSRs, A/T was the most frequent motif (146 loci, 32.1%), whereas C/G was rare (1 locus, 0.2%) (Fig. 3B).

**Fig. 3:**
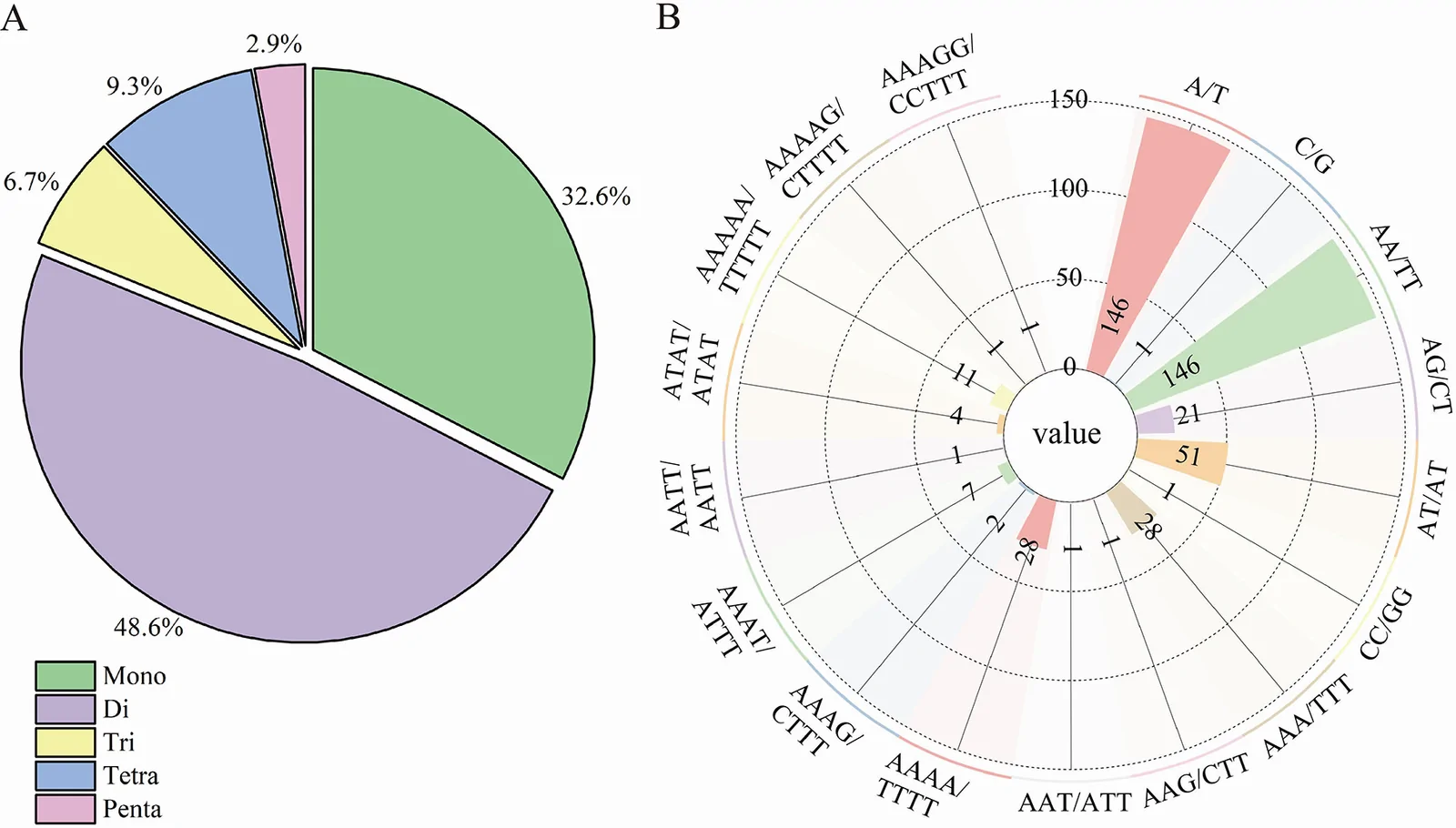
Simple sequence repeats (SSR) identified in the chloroplast genome of *M. nigra*. A. Number of each SSR motif type. B. Proportion of SSR motif types (mono-, di-, tri-, tetra-, penta-, and hexanucleotide repeats).

In the codon usage analysis (Fig. 4), the arginine codon AGA had the highest relative synonymous codon usage (RSCU) value (1.97), followed by the leucine codon UUA (RSCU=1.87). Due to the degeneracy of the genetic code, tryptophan and methionine are each encoded by a single codon, whereas other amino acids are encoded by two or more codons. Among all codons, 30 had RSCU values greater than 1, 32 had values <1, and the codons for tryptophan and methionine each had an RSCU of 1. Codons ending in A or U were markedly more frequent than those ending in G or C, which generally showed RSCU values below 1, indicating a strong codon preference for A/U endings in the chloroplast genome of *M. nigra*.

**Fig. 4:**
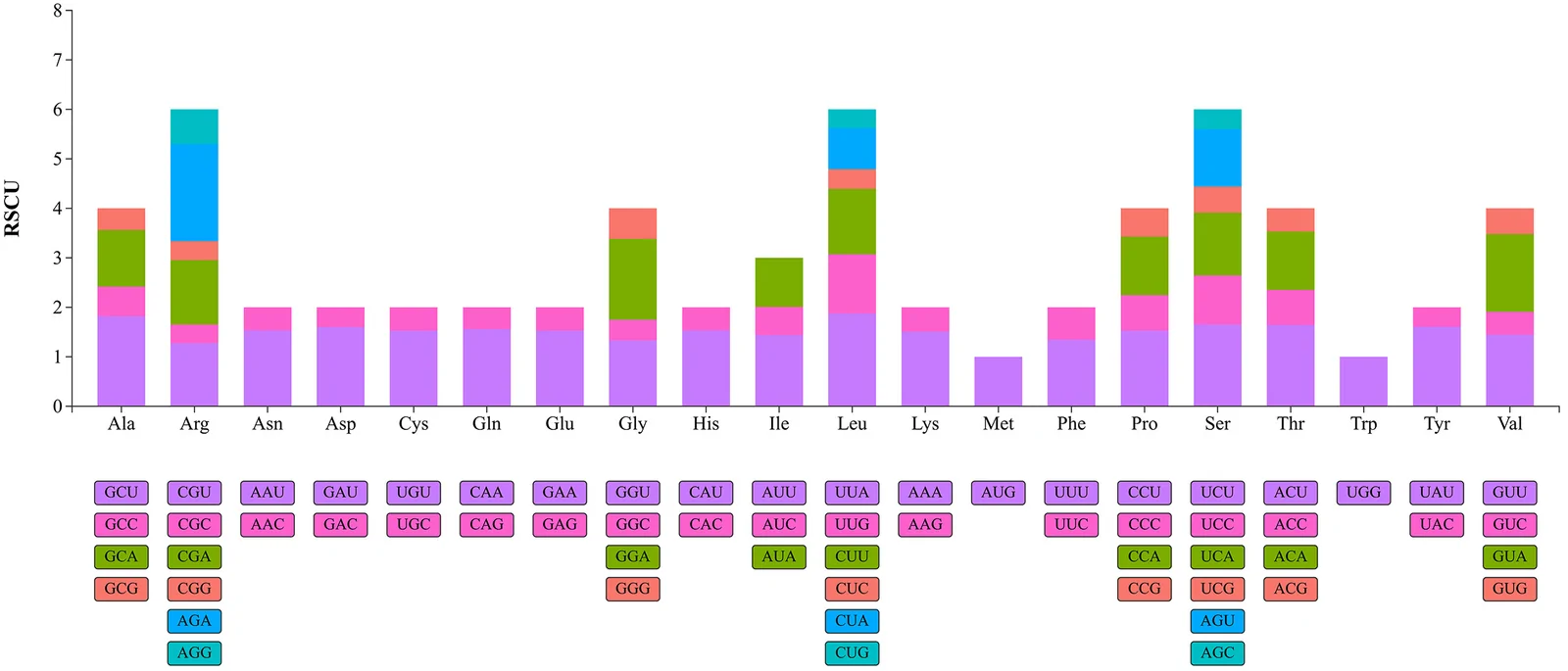
Relative synonymous codon usage (RSCU) value for all codons in the chloroplast genome of *M. nigra*.

Boundary comparisons among the chloroplast genomes of eight *Morus* species revealed the four canonical junctions between the IR and the SC region: LSC/IRb, IRb/SSC, SSC/IRa, and IRa/LSC (Fig. 5). The gene arrangement at these junctions was highly conserved across species. In *M. nigra*, the *rps19* gene is located 1 bp from the SSC/IRb boundary, whereas in the other seven species it is positioned >1 bp away. The *trnH* gene lies 26 bp from the IRa/LSC boundary in *M. notabilis* and 23 bp away in the other seven species. The *ndhF* gene spans the IRb/SSC boundary in all eight species. In addition, *ycf1* crosses the SSC/IRa junction, and its truncated copy in the IR region is considered a pseudogene.

**Fig. 5:**
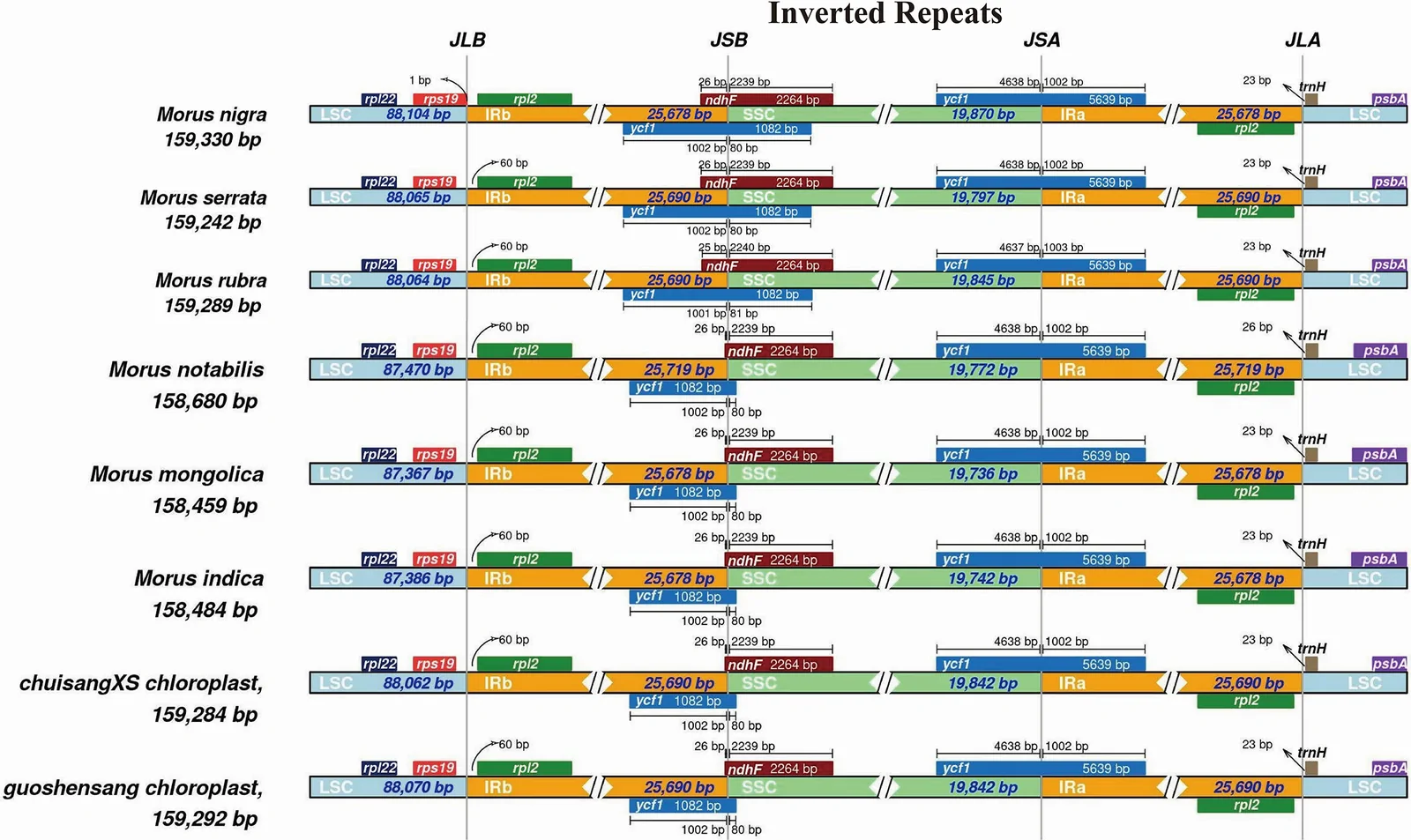
Chloroplast genome boundary analysis of eight *Morus* species.

Phylogenetic analysis based on the chloroplast genomes of *M. nigra* and 26 published Moraceae species revealed that eight *Morus* species formed a monophyletic clade, with *M. nigra* occupying an independent branch (Fig. 6). Overall, this study provides valuable genomic information on *M. nigra* and will facilitate the identification and utilization of this medicinal species.

**Fig. 6:**
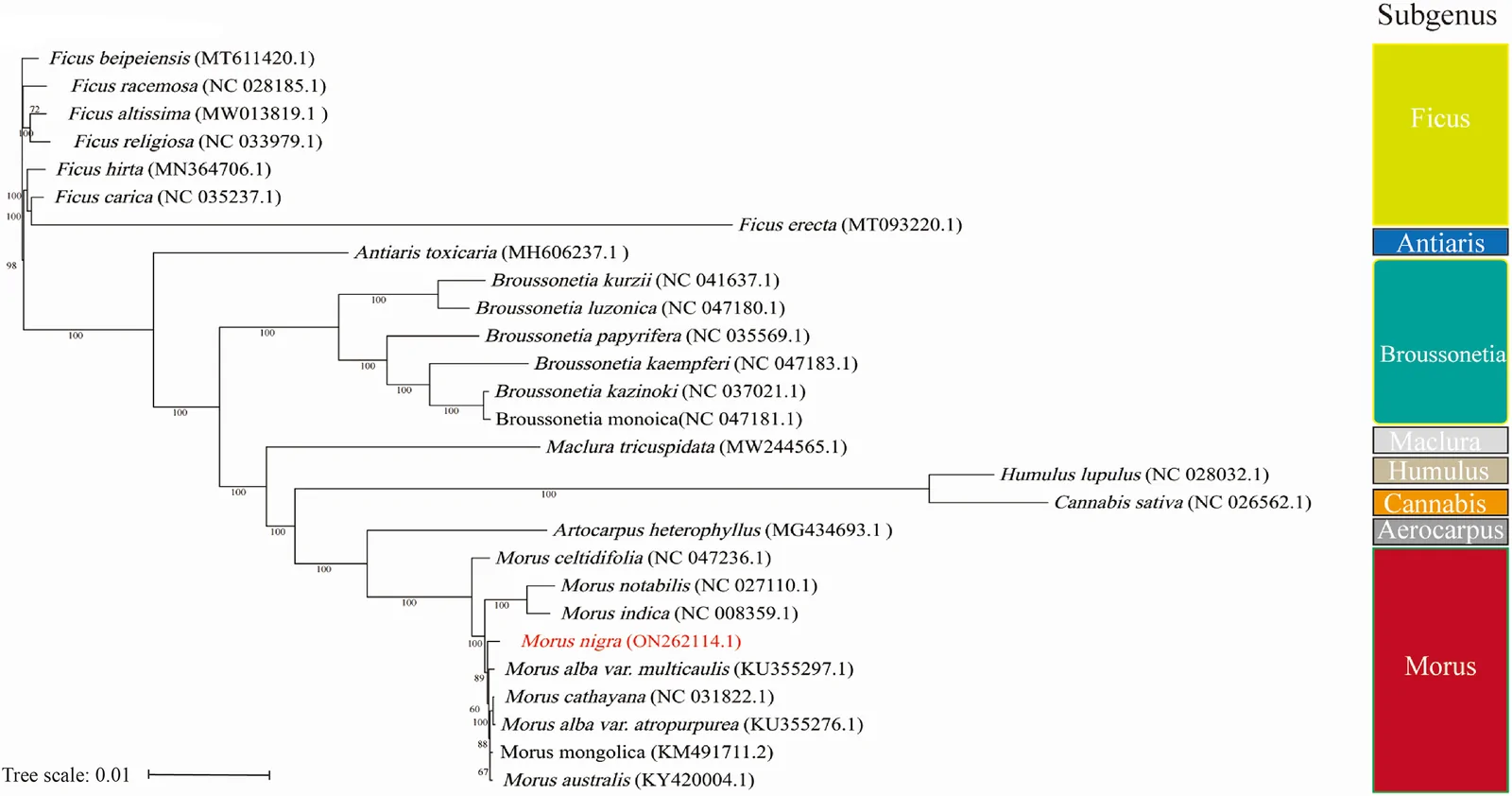
Maximum likelihood phylogenetic tree based on the complete chloroplast genome sequences of 27 Moraceae species. The species sequenced in this study (*Morus nigra*) is marked with a red color.

## 4 Experimental Design, Materials and Methods

### 4.1 Plant materials and DNA extraction

Fresh young leaves of *Morus nigra* were collected from cultivated plants in Chengde, Hebei Province, China (41°2′19.40″N, 117°57′48.10″E). The voucher specimen is preserved in the specimen room of the Sericultural Research Institute of Chengde Medical University (CYYJS01). Total genomic DNA was extracted from leaf tissue using a modified CTAB protocol [12]. DNA quality and quantity were assessed by 1% agarose gel electrophoresis, a Nanodrop spectrophotometer, and a Qubit fluorometer. DNA samples meeting sequencing requirements were sent to Benagen Tech Solutions (Wuhan, China).

### 4.2 Sequencing and sequence analyses

Short-read and long-read libraries were sequenced using the Illumina NovaSeq 6000 and the Oxford Nanopore platform, respectively. Raw sequence data were quality-checked using FastQC v0.11.9 [13]. Short-read and long-read datasets were further filtered using fastp and NanoPack 2, respectively, and only high-quality reads were retained for subsequent analyses. Chloroplast-derived contigs were identified by BLASTn against the chloroplast genome of *M. alba* (GenBank: NC_057087.1) with the parameters ‘-evalue 1e-5 -outfmt 6 -max_hsps 10 -word_size 7 -task blastn-short’. Short-reads and long-reads were then assembled into the chloroplast genome using Unicycler v0.5.0 [14]. Unicycler was selected because chloroplast-derived contigs were first identified by BLASTn alignment against the *M. alba* chloroplast genome, and the hybrid short-read/long-read assembly strategy is well suited for circular organelle genomes. Coding sequences (CDSs) were annotated using Prodigal v2.6.3 (https://www.github.com/hyattpd/Prodigal), while rRNA and tRNA genes were predicted using HMMER v3.1b2 (http://www.hmmer.org) and ARAGORN v1.2.38 (http://www.ansikte.se/ARAGORN), respectively. Manual curation of the annotations was performed using CPGView [15] and Apollo [16].

### 4.3 SSR, RSCU and IR boundary analysis

Simple sequence repeats (SSRs) were identified using the MISA web tool, with minimum repeat numbers of 10, 5, 4, 3, 3, and 3 for mono-, di-, tri-, tetra-, penta-, and hexanucleotide motifs, respectively, and a minimum inter-SSR spacing of 100 bp. The online BioDataTools platform (https://www.biodatatools.top) was used to analyze base composition and codon usage. IRscope (http://genocat.tools/tools/irscope.html) [17] was used to identify and visualize the boundaries of the LSC, SSC, IRa and IRb regions in each species.

### 4.4 Phylogenetic analysis

To determine the phylogenetic position of *M. nigra*, a maximum likelihood phylogenetic analysis was performed using the chloroplast genomes of *M. nigra* and 26 published Moraceae species using MEGA X [18] with 1000 bootstrap replicates under the general time-reversible (GTR) model.

## Limitations

None.

## Ethics Statement

All authors have read and follow the ethical requirements for publication in Data in Brief and confirm that the current work does not involve human subjects, animal experiments, or any data collected from social media platforms.

## CRediT Author Statement

**Binbin Wang:** Data curation, Writing-original draft; **Danyang Wang:** Investigation, Methodology, Project administration; **Hui Wang:** Formal analysis, Validation, Data curation; **Yanxia Gao:** Conceptualization, Funding acquisition, Writing-review & editing*.*

## Data Availability

*Morus nigra* chloroplast, complete genome (Original data) (NCBI); Proportion of SSR motif types and RSCU value for all codons in the chloroplast genome of *Morus nigra* (Reference data).
